# Why we should rethink military metaphors in cancer

**DOI:** 10.36416/1806-3756/e20250109

**Published:** 2025-05-21

**Authors:** Gustavo Faibischew Prado, Thais Azzi Gonçalves

**Affiliations:** 1. Comitê Científico de Câncer de Pulmão, Sociedade Brasileira de Pneumologia e Tisiologia, Brasília (DF) Brasil.; 2. Departamento de Doenças Respiratórias, Rede D’Or, São Paulo (SP) Brasil.; 3. Coordenação de Saúde Oral, Unidade F. Abreu, São Paulo (SP) Brasil.

There is something both seductive and treacherous about military metaphors when we talk about diseases. From the moment of diagnosis, the patient is often enlisted-without choice-into a personal war. We tell them they must be strong, that they need to fight, that giving up is not an option. If the disease progresses, “the enemy was relentless”; if they pass away, “they lost the battle.” These expressions are so deeply embedded in our medical, journalistic, and everyday language that we rarely stop to question them. But we should.

“*It took me years to understand why it bothered me to be called a ‘warrior,’ especially when, in moments of vulnerability, comments reinforced this ‘battle.’ This metaphor imposes an unnecessary burden and fuels guilt that ignores the complexity of the illness experience.”*

War is a setting of cruelty and sacrifice; and our perceptions of these harrowing events, especially those from the early 20th century to today, show us unequivocally that there is nothing romantic about them.

These metaphors rely on two premises: there is an enemy (the disease) and a clear objective-victory. However, diseases are not adversaries that can be vanquished through willpower or a positive attitude, and patients are not soldiers who fail when treatment does not yield the desired effect.

“*During treatment, I wasn’t fighting against something; I was simply living one day at a time. Talking about a fight gives the impression that sheer willpower is enough to win, but disease doesn’t work that way.*”

Cancer treatment is frequently described as a battle in news reports and social media. For example: “On December 5, 2015 (...) Marília Pêra lost her battle against lung cancer, which she had been fighting for two years.”^1^

This illustrates how the disease is portrayed as an invading army. However, unlike this well-intentioned depiction, cancer is a complex phenomenon that arises from our own bodies, the result of biological processes that are sometimes beyond modification. Its progression is far more influenced by factors beyond our control than by the patient’s emotional resilience.

## THE BURDEN OF BLAME

The problem with these metaphors is not merely theoretical. In my daily interactions with patients, I see how they impose an immense emotional burden. They reduce and hijack our discourse, which should be compassionate and understanding, into a motivational speech straight out of a self-help seminar. This distances us from patients, deafens our listening, and invalidates their suffering. Worse still, it shifts the unfathomable weight of illness onto the shoulders of this so-called “soldier.” If treatment is not well-tolerated, if it fails to halt disease progression, if symptoms become unbearable-what does that imply? That the patient was not strong enough? That they lacked courage? That they were not a true warrior? This narrative subtly suggests that the outcome of the disease is proportional to individual effort, leading patients to feel like failures in a situation that was never under their control.

This is particularly cruel for cancer patients. For many families, seeing a loved one as a warrior may be a way to make sense of immeasurable suffering, to name the unnamable, to impose order on chaos. But this very metaphor can place an unbearable weight on the patient. What if they can’t take it anymore? What if they cry in pain? What if they no longer want to be “strong”? The idea that “giving up” equates to surrender creates a cycle of anguish for both patients and their loved ones.

## ILLNESS IS NOT A BATTLE, AND THE PATIENT IS NOT A SOLDIER

The way we talk about health shapes how we approach it. The concept of a “war on cancer” is not just a linguistic phenomenon-it influences public policy, directs campaigns, and affects medical decision-making. Since Nixon declared his “war on cancer” in the 1970s,^2^ investment in oncology research has grown significantly, and the military vocabulary has persisted. The unintended consequence is that cancer has come to be seen as something to be “eradicated,” and the patient as someone who must resist at all costs.

“*When facing my second recurrence, the inevitable question arose: had I not fought hard enough? This sense of guilt, the suggestion that something more could have changed the outcome, only deepened the pain of difficult moments.*”

But romanticizing war, idealizing death, and glorifying the fallen hero are nothing new.^3^ These themes are present in Homeric verses, such as Hector’s death in Troy. The Greek epic tradition exalted heroic death in war as the pinnacle of existence-a sacrifice worthy of eternal honor and glory. Yet even within the *Iliad* and the *Odyssey*, this ideal was challenged. Achilles, centuries later, in the underworld, shatters this romanticized vision, declaring that he would rather be a living farmer than a king among the dead.^4^ This cult of war and sacrifice still echoes in the military metaphors we use to describe illness. But does a patient need to be a warrior? Does illness have to be a battle? Among the pain, the fears, and the many uncertainties, perhaps what we need is not a call to arms but a more sensitive approach and a discourse that embraces rather than imposes struggle.

And what happens when we acknowledge that some diseases cannot be defeated-only managed? That living with a chronic condition is not a failure? That accepting the limits of treatment is not surrender but a legitimate form of care?

## METAPHORS MATTER

This does not mean we must eliminate all metaphors-they are part of how we make sense of the world. But perhaps we can choose better ones.

If we insist on comparing illness to war, we will always seek winners and losers. Instead, we could view this challenge as a *journey*-with difficult and lighter moments, with uncertain and unexpected paths, but without a single finish line or a predetermined outcome. The experience of illness is already a difficult road; it does not need to be a battlefield. The journey of treatment should allow suffering to be expressed, validated, and acknowledged. It should enable the patient to find support in care and grant them permission not to be always positive or upbeat.

“*If we compare illness to war, cancer does not play fair. There is no guaranteed strategy, and often it is an endless battle that demands adaptation, coexistence, and management. Living with disease does not mean losing-not every day requires us to be soldiers or heroes.”*

And all of us, as participants in a care network, can take on other roles: supporters, partners, and friends.

Because yes, we fall ill; and yes, one day, we will die. But life, death, and everything beautiful-and challenging-that happens in between can (and should) be lighter.

After all, no one should die thinking they lost.
